# Airborne transmission of invasive fusariosis in patients with hematologic malignancies

**DOI:** 10.1371/journal.pone.0196426

**Published:** 2018-04-26

**Authors:** Maria Luiza Moretti, Ariane Fidelis Busso-Lopes, Cibele Aparecida Tararam, Renato Moraes, Yasunori Muraosa, Yuzuru Mikami, Tohru Gonoi, Hideaki Taguchi, Luzia Lyra, Franqueline Reichert-Lima, Plínio Trabasso, Gerrit Sybren de Hoog, Abdullah Mohammed Said Al-Hatmi, Angelica Zaninelli Schreiber, Katsuhiko Kamei

**Affiliations:** 1 Department of Internal Medicine, Faculty of Medical Sciences, University of Campinas, Campinas, Sao Paulo, Brazil; 2 Brazilian Biosciences National Laboratory, Campinas, Brazil; 3 Medical Mycology Research Center, Chiba University, Chiba, Japan; 4 Department of Clinical Pathology, Faculty of Medical Sciences, University of Campinas, Campinas, Sao Paulo, Brazil; 5 CBS-KNAW Fungal Biodiversity Centre, Utrecht, The Netherlands; 6 Institutes of Biodiversity and Ecosystem Dynamics, University of Amsterdam, Amsterdam, The Netherlands; 7 Basic Pathology Department, Federal University of Paraná State, Curitiba, Paraná, Brazil; 8 Biology Department, Faculty of Science, King Abdulaziz University, Jeddah, Saudi Arabia; 9 Directorate General of Health Services, Ibri Hospital, Ministry of Health, Muscat, Oman; Soonchunhyang University, REPUBLIC OF KOREA

## Abstract

From 2006 to 2013, an increasing incidence of fusariosis was observed in the hematologic patients of our University Hospital. We suspected of an environmental source, and the indoor hospital air was investigated as a potential source of the fungemia. Air samplings were performed in the hematology and bone marrow transplant (BMT) wards using an air sampler with pre-defined air volumes. To study the molecular relationship among environmental and clinical isolates, 18 *Fusarium* spp. recovered from blood cultures were included in the study. DNA sequencing of a partial portion of *TEF1α* gene was performed for molecular identification. Molecular typing was carried out by multi-locus sequence typing (MLST) using a four-gene scheme: *TEF1α*, rDNA, *RPB1* and *RPB2*. One hundred four isolates were recovered from the air of the hematology (n = 76) and the BMT (n = 28) wards. *Fusarium* isolates from the air were from five species complexes: *Fusarium fujikuroi* (FFSC, n = 56), *Fusarium incarnatum-equiseti* (FIESC, n = 24), *Fusarium solani* (FSSC, n = 13), *Fusarium chlamydosporum* (FCSC, n = 10), and *Fusarium oxysporum* (FOSC, n = 1). Fifteen *Fusarium* isolates recovered from blood belonged to FSSC, and three to FFSC. MLST identified the same sequence type (ST) in clinical and environmental isolates. ST1 was found in 5 isolates from blood and in 7 from the air, both identified as FSSC (*Fusarium petroliphilum*). STn1 was found in one isolate from blood and in one from the air, both identified as FFSC (*Fusarium napiforme*). *F*. *napiforme* was isolated from the air of the hospital room of the patient with fungemia due to *F*. *napiforme*. These findings suggested a possible clonal origin of the *Fusarium* spp. recovered from air and bloodcultures. In conclusion, our study found a diversity of *Fusarium* species in the air of our hospital, and a possible role of the air as source of systemic fusariosis in our immunocompromised patients.

## Introduction

Filamentous fungi of the genera *Fusarium* are ubiquitous in the environment and are found in the soil, water, and air [1]. *Fusarium* species are primarily plant pathogens, and produce toxins that can cause food poisoning[2]. *Fusarium* can also infect humans and cause localized or disseminated diseases depending on the predisposing factors and the immunological status of the host [3, 4]. Keratitis[2] and onychomycosis[5] are the most common infections in the immunocompetent hosts, and the infections may also occur because of trauma or by the use of contaminated contact lens[6, 7].

In the immunocompromised patients, especially those with hematologic malignancies and recipients of allogeneic hematopoietic stem cell transplantation (HSCT), *Fusarium* can disseminate in the organism causing a systemic and invasive infection (fusariosis) [3, 8, 9]. *Fusarium solani* is the most frequent species involved in fusariosis (50% of cases), followed by *Fusarium oxysporum* (20%) and *Fusarium verticillioidis* and *Fusarium moniliforme* (10% each). In Brazil, *Fusarium* is the leading cause of invasive mold infections, followed by *Aspergillus*, with an overall incidence of 6 cases per 1,000 HSCTs[3, 10, 11]. *Fusarium* spp. may show an antifungal susceptibility profile marked by high level of resistance; however some isolates can be susceptible *in vitro* to amphotericin B, voriconazol[12, 13], notwithstanding, the mortality rate iof disseminated fusariosis may exceed 75%.

For severely immunocompromised patients, hospitalization in a controlled environment has been recommended, such as in private rooms equipped with high-efficiency particulate air (HEPA) filters and positive airflow system. Several studies evaluated the effectiveness of HEPA filters in preventing or reducing invasive aspergillosis in hematologic and oncologic patients[14, 15], but did not evaluate other filamentous fungi, such as *Fusarium*.

From 2006 to 2013, 34 patients from the hematology and the bone marrow transplant (BMT) wards were diagnosed with invasive fusariosis in our University Hospital, with an incidence density during this period ranging from 0.18 to 2.4 per 1,000 patients-day (incidence density in 8 years: 0.82 cases per 1,000 patients-day). Fusariosis, in our hospital, was surprisingly higher than most hospitals worldwide[11] raising the hypothesis that an environmental source could be implicated in the high incidence of fusariosis. Therefore, we studied the air as a potential environmental source for invasive fusariosis by comparing the genetic relationship of *Fusarium* isolates obtained from the environment, as well as the ones obtained from blood cultures.

## Materials and methods

### Study location

This study was performed at the Clinical Hospital of the University of Campinas, Campinas, Sao Paulo, Brazil. It is a 419-bed tertiary-care university hospital and is the referral hospital for all major medical services in an area of 3,000,000 inhabitants. The BMT ward has 7 rooms with 9 beds, HEPA filters and positive pressure airflow. The hematology ward has 9 rooms with 11 beds and no controlled air. All patients with hematologic malignancies were hospitalized in this ward and patients that underwent HSCT were hospitalized in the BMT. As we had an increasing number of systemic fusariosis in patients with hematologic malignancies, we assumed that we were having an outbreak with a potential source that needed a prompt investigation by the Infection Control Team and the Mycology Laboratory. The Infection Control Division performed the air samplings without consulting the University Ethical Committee, as that investigation of potential sources of outbreaks is mandatory.

### Clinical isolates

Eighteen isolates of *Fusarium* obtained from 15 patients with hematologic diseases, between 2007 and 2013, were included in this study. *Fusarium* species were isolated from blood cultures by using the Bact/ALERT (BioMérieux, France) and subsequent morphology evaluation in Sabouraud Dextrose Agar medium (Difco, USA)[16]. Fifteen isolates were from individual patients and three isolates were from three different blood samples withdrawn from the same patient. All samples were collected for routine diagnostic exams and no clinical information was collected from the patients’ records. The clinical isolates numbers: 917, 952, 1192, 1549, 1601, 1603, 1631, 1750, 2020, 916, 1103,1202, 1207, 1372 and 1554 were isolated from patients hospitalized from July 2007 to July 2011, and they were stored in the Mycology Laboratory Culture Collection. As these isolates belonged to a Culture Collection, there was no need for ethical approval. The isolates number 2008, 2009 and 2010 were from patients that participated in the Project CAAE 0870.0.146.000–11 approved by the Ethical Committee Decision No. 964/2011 (Principal investigators: M. de Sousa and P. Trabasso)[17].

### Air sampling

The air samplings were performed from March 2012 to March 2013 at the hematology and BMT wards. The samplings were performed during summer, autumn, and spring seasons. The air was collected by the air sampler Bio Samp Model MBS 1000D (Yotsubishi Corp., Japan) in a selective culture medium for *Fusarium* modified by Mikami Y., Chiba University, Japan[18]. The volume of 1,000 L and 500 L of air was collected from the BMT and hematology wards, respectively. All the samples were taken approximately 1.5 m above the floor and the air was sampled three times in the same room and once in the bathroom. Air isolates were inoculated in Sabouraud Dextrose Agar medium (Difco, USA). Temperature and humidity during sampling were also recorded. The plates were incubated at 37°C for 15 days and fungi with micro and macro morphology resembling *Fusarium* spp. and the strains for working stock were stored in distilled water[16, 19].

### Molecular identification

#### DNA extraction

Strains were transferred to Sabouraud Dextrose Agar medium (Difco, USA) and incubated at room temperature for 7 days. DNA was extracted using the QiaAmp DNA Mini Kit (Qiagen, USA), according to the manufacturer’s instructions.

#### PCR reactions

PCR was performed using specific primers in order to amplify four genes fragments chosen for *Fusarium* identification and MLST: *TEF1α* (translation elongation factor—1α), rDNA (ribosomal DNA), *RPB1* (RNA polymerase largest subunit) and *RPB2* (RNA polymerase second largest subunit). The position and sequences of the primers used in this study are described in **Table 1**. PCR was performed using PCR Master Mix (Promega, USA). PCR reactions were incubated in a Veriti 96 well Thermal Cycler (Applied Biosystem, USA) under the following conditions: 2 min of initial denaturation at 98°C, 40 cycles of DNA denaturation at 98°C for 30 s, primer annealing temperature varying according to the target gene for 30 s, elongation at 72°C for 1 min and a final elongation step at 72°C for 5 min. PCR products were verified by electrophoresis in a 2% agarose gel, 100 v for 30 min. PCR products were purified with ExoSAP-IT for PCR Product Clean-up (Affymetrix USB, USA) prior to sequencing analysis.

**Table 1:** Primers used for sequencing of clinical and environmental *Fusarium* isolates.

Gene	Protein	Primer		Reference
		Name	Sequence (5' - 3')^a^	
*TEF1α*	Translation elongation factor 1 alpha	HS392	TCAAAATGGGTAAGGA(A/G)GACAAGAC	[20, 21]
		HS393	GCCTGGGA(A/G)GTACCAGT(C/G)ATCATGTT	[20, 21]
		EF11	GTGGGGCATTTACCCCGCC	[21]
		EF21	GAGTGGCGGGGTAAATGCC	[21]
rDNA	Ribosomal DNA	ITS4	TCCTCCGCTTATTGATATGC	[23]
		ITS5	GGAAGTAAAAGTCGTAACAAGG	[24]
		NL1	GCATATCAATAAGCGGAGGAAAAG	[23]
		NL4	GGTCCGTGTTTCAAGACGG	[24]
*RPB1*	RNA polymerase largest subunit	Fa	CAYAARGARTCYATGATGGGWC	[25]
		F5	ATGGGTATYGTCCAGGAYTC	[25]
		F7	CRACACAGAAGAGTTTGAAGG	[25]
		F8	TTCTTCCACGCCATGGCTGGTCG	[25]
		R8	CAATGAGACCTTCTCGACCAGC	[25]
		G2R	GTCATYTGDGTDGCDGGYTCDCC	[25]
		R9	TCARGCCCATGCGAGAGTTGTC	[25]
		F2	GATGGGATCGBGCHTTYGTCA	This study
		F1c	GACTGGTTCAAGCATGACTACGAAT	This study
		F1e	CGACAAGTGCGACAGATTAACAAGG	This study
*RPB2*	RNA polymerase second largest subunit	6F	TGGGGKWTGGTYTGYCCTGC	[26]
		5F2	GGGGWGAYCAGAAGAAGGC	[24]
		7cR	CCCATRGCTTGYTTRCCCAT	[24]
		7cF	ATGGGYAARCAAGCYATGGG	[24]
		11aR	GCRTGGATCTTRTCRTCSACCC	[24]
		40R	AGCTTGCGTCCAGTATGACC	[23]
		40-2F	CAAAAACCTCTGGCGACAAC	[23]
		2F	ATTTGCATGACKCCNGARGATC	This study
		2R	ACTRCTCTGGTTCATAATGACGGAA	This study

bp: base pairs.

^a^ Y: C or T; W: A or T; R: A or G; K: G or T; S: G or C; D: A, G or T; B: G, T or C; H: A, C or T.

#### DNA sequencing for identification

A partial portion of *TEF1α* was sequenced with the BigDye Terminator reagent kit (Applied Biosystems, USA) in an ABI Prism 3,100 Genetic Analyzer (Applied Biosystems, USA) using HS392, HS393, EF11 and EF21 primers [20, 21] (**Table 1**). DNA sequences were edited and assembled by Sequencher version 5.2.4 (Gene Codes, USA). For identification, a homology search for the sequences of *TEF1α* gene was done using the BLAST tool of the NCBI database (GenBank), the database FUSARIUM-ID (*http://isolate.fusariumdb.orgl/*), and the *Fusarium* CBS database (*http://www.cbs.knaw.nl/fusarium*). To confirm the identity of our *Fusarium* species, we evaluated their position with maximum likelihood (ML) method and a tree of *TEF1α* analysis was constructed. In these analyses, our sequences, together with sequences retrieved from GenBank and CBS database, were analyzed. Consensus sequences were computed with SeqMan from the Lasergene package (DNA Star, USA). Sequences were aligned with the program MAFFT (*www*.*ebi*.*ac*.*uk/Tools/msa/mafft/*), followed by manual adjustments with MEGA 6 [22] and BioEdit v7.0.5.2.

#### Multi Locus Sequencing Typing (MLST) for isotyping

*Fusarium* isolates found in air and blood from to the same species or species complex were submitted to MLST. Portions of the following four genes fragments were chosen for MLST: *TEF1α* (598 bp), rDNA (1,029 bp), *RPB1* (2,705 bp) and *RPB2* (1,750 bp) (**Table 1; S1 Fig**). The number of loci used for MLST was based on the previous studies of Scheel et al [23]which include *TEF1α*, rRNA and *RPB2* and O’Donnell et al [27] that used *TEF1α*, *RPB1* and *RPB2* for phylogenetic analysis. We combined the loci described by both authors to generate a 4-loci scheme with more robust genetic typing. These four portions were sequenced in an ABI Prism 3,100 Genetic Analyzer (Applied Biosystems, USA) using primers described before[20, 21] and additional designed primers (**Table 1**). DNA sequences were edited and assembled by Sequencher version 5.2.4 (Gene Codes, USA). Sequences for all genes (6,082bp) were aligned by Clustal W tool and followed manual adjustments with MEGA6[22]. Numbers were assigned to each allelic variant and combined in order to generate a unique sequence type (ST) for every *Fusarium* isolate. The sequence data obtained in this study was deposited in GenBank and the accession numbers are listed in **S1 Table.**

## Results

### Air sampling and climatic conditions

We performed nine air samplings from 2012 to 2013. Five air samplings were positives for the recovery of *Fusarium* spp. and four samplings resulted negatives (**S2 Table**). One hundred and four isolates were recovered from hematology (n = 76; 73.1%) and BMT units (n = 28; 26.9%). The median temperature during the sampling days was 30.4 ± 3.68 (°C) and the median humidity varied from 49.0 ± 17.2 to 72.0 ± 15.4 (^o^C). No relationship was found between the dates of air samplings, climate conditions, and season of the year, and the number of *Fusarium* species isolated from the hospital air.

### Identification by *TEF1α* sequencing

**Fig 1:**
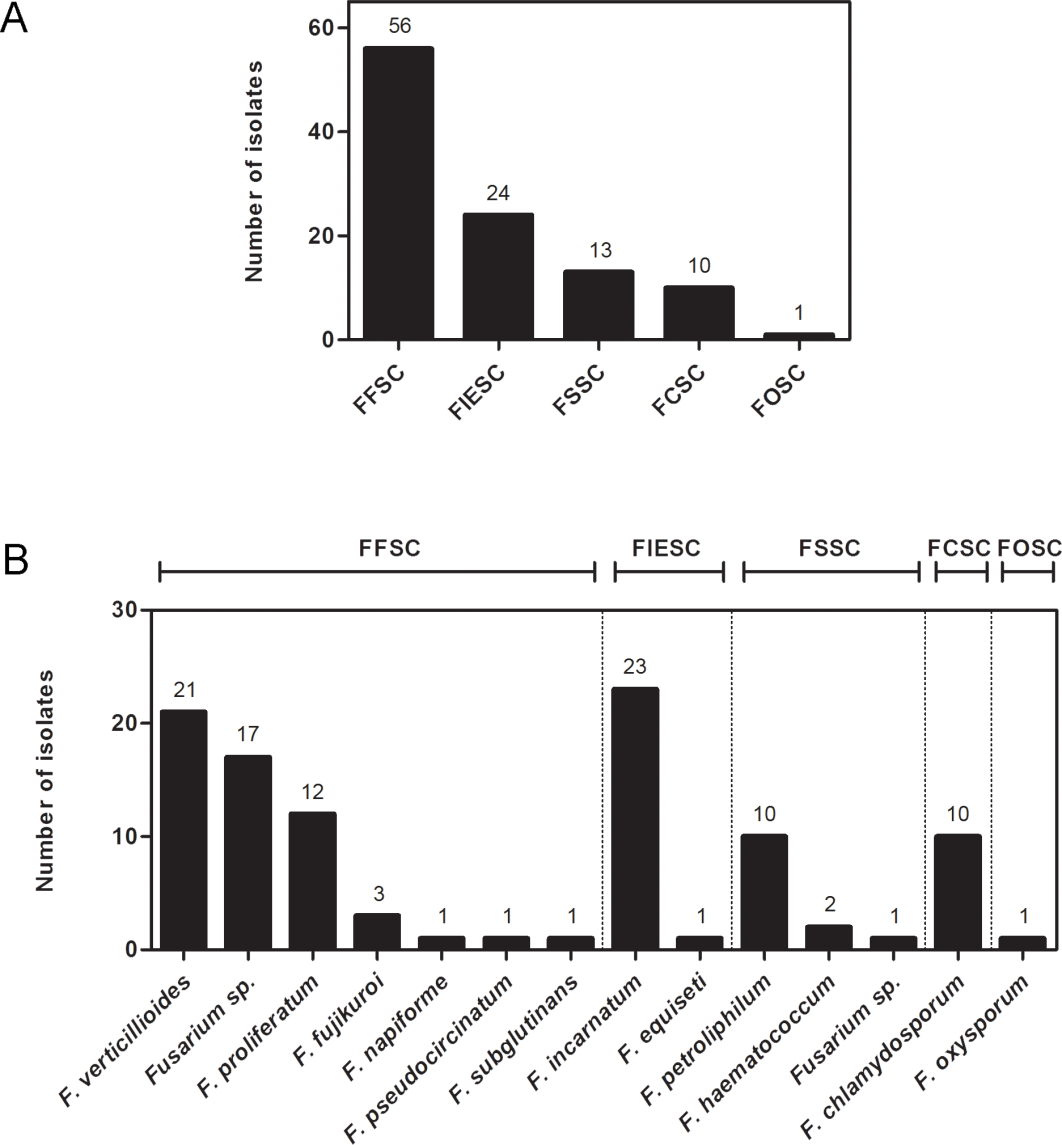
Molecular identification of *Fusarium* species isolated from hospital air samplings. (A) and (B) shows *TEF1α* DNA sequencing classification in species complex and species, respectively. The number of isolates is shown above each bar. FCSC: *F*. *chlamydosporum* species complex; FFSC: *F*. *fujikuroi* species complex; FIESC: *F*. *incarnatum-equiseti* species complex; FOSC: *F*. *oxysporum* species complex; FSSC: *F*. *solani* species complex.

The DNA sequencing of a portion of *TEF1α* gene was performed for air (n = 104) and clinical (n = 18) *Fusarium* isolates. Results of phylogenetic analysis of the 104 strains from air assigned 86 strains to species level, belonging to five species complexes: *F*. *solani* (FSSC), *Fusarium fujikuroi* (FFSC), *Fusarium oxysporum* (FOSC), *Fusarium incarnatum-equiseti* (FIESC) and *Fusarium chlamydosporum* (FCSC) **(Fig 1, S2–S4 Figs)**. The most common species of FFSC isolated from air was *Fusarium verticillioides* (n = 21 isolates), followed by *Fusarium proliferatum* (n = 12), *F*. *fujikuroi* (n = 3), *Fusarium napiforme* (n = 1), *Fusarium pseudocircinatum* (n = 1), and *Fusarium subglutinans* (n = 1). The species of FSSC isolated from air were *Fusarium petroliphilum* (n = 10) and *Fusarium haematococcum* (n = 2). We also recovered 24 isolates of FIESC (23 *Fusarium incarnatum* and 1 *Fusarium equiseti*), 10 FCSC (*F*. *chlamydosporum*), and 1 FOSC (*F*. *oxysporum*). Eighteen isolates from the air were not assigned to species level: FFSC (n = 17), and FSSC (n = 1), and formed well-supported monophyletic branches suggesting that further phylogenetic work is necessary for species delimitation and description for these isolates **(Fig 1, S2–S4 Figs)**.

**Fig 2:**
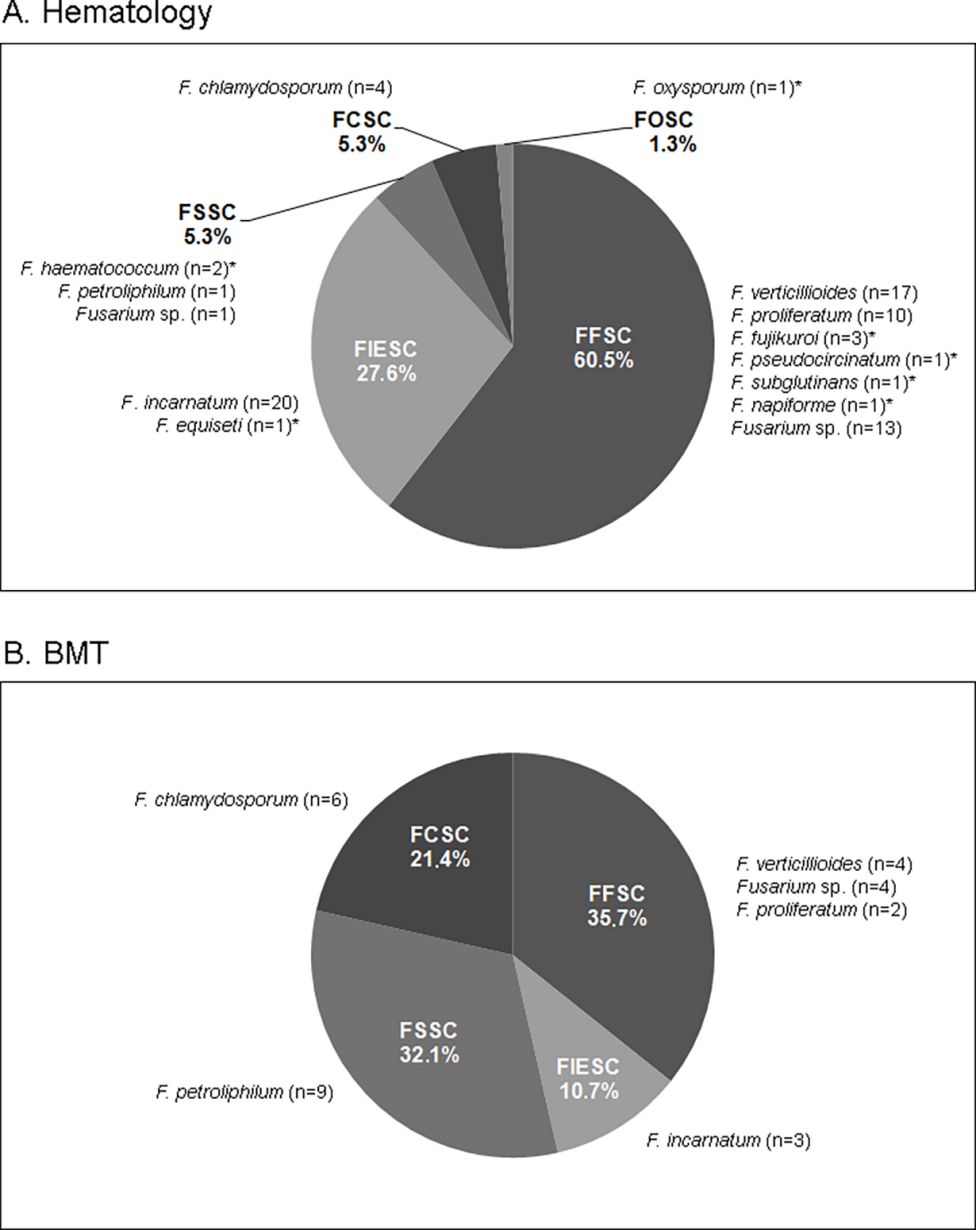
Distribution of *Fusarium* species isolated from hospital air. The frequency of each species complex in the hematology (A, n = 76) and BMT (B, n = 28) wards is shown. The species identified for each complex is presented outside the graphs. The species found exclusively in hematology unit are marked with (*). FCSC: *F*. *chlamydosporum* species complex; FFSC: *F*. *fujikuroi* species complex; FIESC: *F*. *incarnatum-equiseti* species complex; FOSC: *F*. *oxysporum* species complex; FSSC: *F*. *solani* species complex.

Our results also showed that air isolates that belong to FFSC were predominant in the hematology unit (n = 46 isolates, 60.5%), followed by FIESC (n = 21, 27.6%), FSSC (n = 4, 5.3%), FCSC (n = 4, 5.3%), and FOSC (n = 1, 1.3%) **(Fig 2)**. In the BMT, FFSC isolates were predominant (n = 10, 35.7%), followed by FSSC (n = 9, 32.1%), FCSC (n = 6, 21.4%), and FIESC (n = 3, 10.7%).

Among 18 *Fusarium* isolates from blood cultures, 15 belonged to FSSC and three isolates to FFSC. The phylogenetic analysis classified the clinical isolates into *F*. *petroliphilum* (FSSC, n = 9 isolates), *F*. *keratoplaticum* (FSSC, n = 5), and *F*. *napiforme* (FFSC, n = 3) (**S2 and S3 Figs)**. The phylogenetic tree also presented the monophyly of one undefined species of FSSC (isolate 1554) (**S3 Fig)**. Detailed information about the *Fusarium* isolates (isolation date, ward, room, and sequencing identification) is shown in **S1 Table.**

### Molecular typing of clinical and air isolates

**Fig 3:**
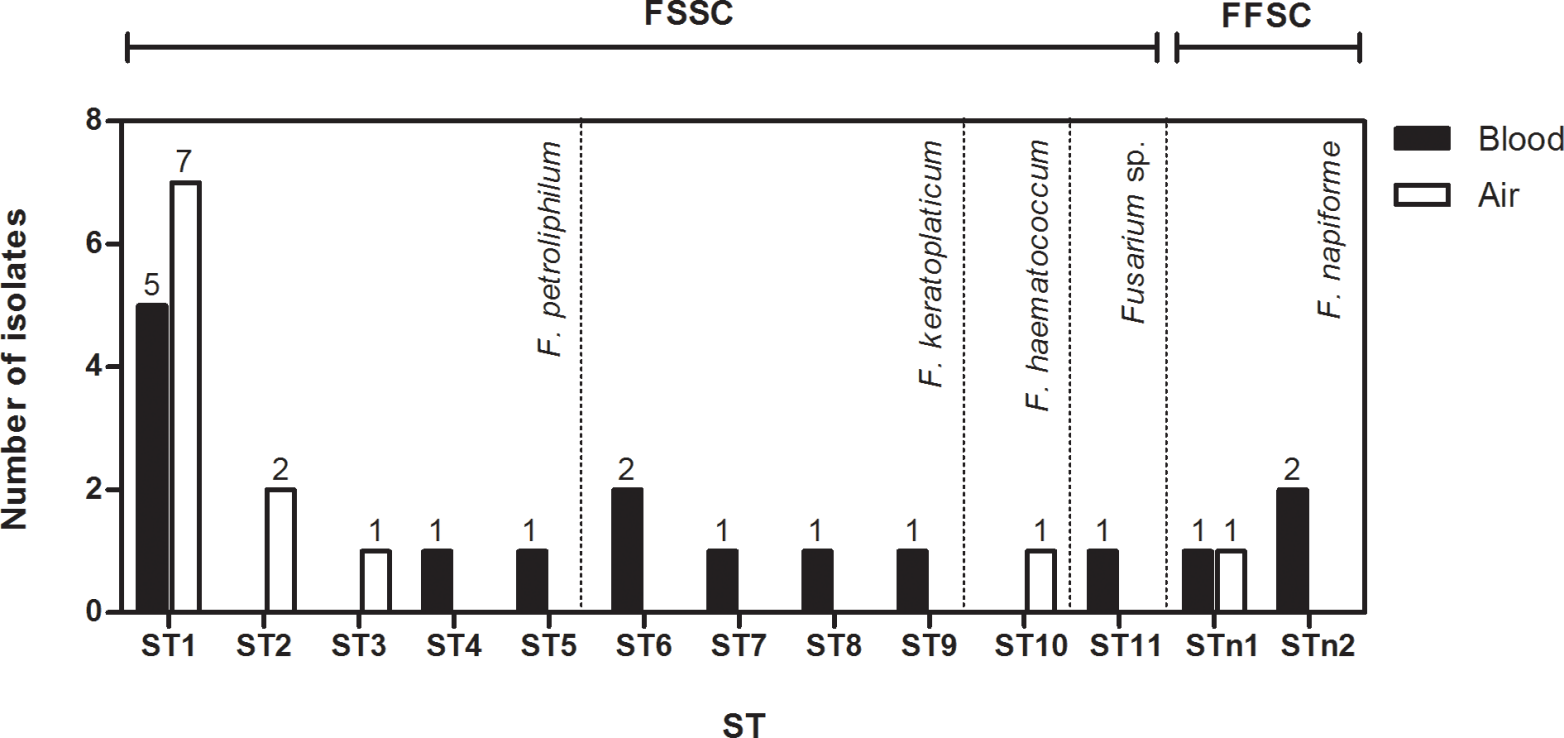
Sequence types (ST) determined by sequencing of portions of the genes *TEF1α*, rDNA, *RPB1* and *RPB2* for *Fusarium* species isolated from air and blood. The number of samples with each ST is shown above the bar. FSSC: *F*. *solani* species complex. FFSC: *F*. *fujikuroi* species complex. ST: sequence type.

*Fusarium* isolates belonging to FSSC and *F*. *napiforme* (FFSC) recovered from blood (16 isolates) and from the air (12 isolates) were submitted to clonal origin analysis (MLST). Thirteen distinctive STs were determined based on the four-locus dataset (6,082 bp): 2 STs for *F*. *napiforme* isolates (STn1 and STn2) and 11STs for FSSC (ST1 to ST11) **(Fig 3)**. Eight STs were assigned only for blood *Fusarium* isolates (ST4 to ST9, ST11 and STn2), and 3 STs were found exclusively in isolates from the air (ST2, ST3 and ST10).

ST1 and STn1 were found in clinical and environmental isolates and determined a possible clonal origin between blood and air *Fusarium*. ST1 was found in 5 isolates of *F*. *petroliphilum* (FSSC) from blood that were recovered from hospitalized patients from 2007 to 2013 (BMT: 1 isolate, Hematology: 4 isolates), and 7 isolates of *F*. *petroliphilum* recovered from the air in 2012 (BMT: 6 isolates, Hematology: 1 isolate) **(Table 2).** STn1 (*F*. *napiforme*) was detected in one isolate from blood culture of a patient that was hospitalized in the hematology unit and in one isolate from the air. Both were isolated in 2013 from the same room during the time that the patient was hospitalized and showed a genetic relationship using MLST.

**Table 2:** Detailed information about ST1 (*F*. *petroliphilum—*FSSC) and STn1 (*F*. *napiforme—*FFSC) isolates.

Sample	Source	Isolation date	Ward
**ST1 (*F*. *petroliphilum—*FSSC)**			
952	Blood	11/28/07	Hematology
1196	Blood	03/29/08	BMT
1549	Blood	12/09/10	Hematology
1750	Blood	06/03/11	Hematology
2020	Blood	06/26/13	Hematology
F16	Air	03/05/12	Hematology
F17-1	Air	03/29/12	BMT
F17-2	Air	03/29/12	BMT
F50	Air	10/10/12	BMT
F51	Air	10/10/12	BMT
F52	Air	10/10/12	BMT
F54	Air	10/10/12	BMT
**STn1 (*F*. *napiforme*- FFSC)**			
2008	Blood	11/27/13	Hematology
F111	Air	03/21/13	Hematology

FSSC: *F*. *solani* species complex; FFSC: *F*. *fujikuroi* species complex; BMT: bone marrow transplant ward.

## Discussion

*Fusarium* species are ubiquitous in the environment, whose spores can easily be carried by wind and rain, causing transmission and subsequent infection to humans. Our study showed, for the first time, a genetic relationship between *Fusarium* species isolated from indoor hospital air with the ones recovered in blood cultures of hematologic patients, suggesting that the air may be a potential source for fusariosis.

Air samplings were performed during one year in BMT and hematology units in our Clinical Hospital. The Clinical Hospital is located in an area surrounded by farms of soy and canola plantations, which may be a potential source of the *Fusarium* isolated in the hospital air once the species are mainly plant pathogens[1]. The hospital is placed on an area of tropical climate with dry winters and very warm rainy summers. Although climate conditions have been previously associated with the onset of crop disease by *Fusarium*[28, 29], the association between the weather and the development of fusariosis is poorly known. A peak of *Fusarium* spores count isolated from indoor hospital and outdoor air in Houston, Texas, USA, was found in the rainy summer of 1988 and 1989[30]. This peak correlated with the seasonal clustering of cases of fusariosis. However, we could not find a correlation between temperature or humidity and the frequency of *Fusarium* isolates recovered in the hospital air.

The recovery of *Fusarium* isolates in the BMT rooms might suggest that the laminar airflow systems were not sufficiently protecting our patients. As the hematology unit does not have controlled air, the higher number of isolates, and the species diversity of *Fusarium* might represent the outside air condition. We presumed that the recovery of a substantial amount of *Fusarium* isolates from the air could be due to the modified selective culture medium used for air sampling[18]. In most studies, Sabouraud Agar formulations were used in the air samplers, and as *Aspergillus* and others filamentous fungi are more prevalent in the air, they were first recovered than *Fusarium* in environmental surveillances[15, 31].

The identification of *Fusarium* to complex or species levels is very important not only to select an appropriate antifungal agent but also to clarify the epidemiology. In this study, we applied *TEF1α* DNA sequencing for identification of the genus *Fusarium* for strains recovered from the air (n = 104) and blood (n = 18). The identification of members of FSSC is of special importance because this complex is responsible for about 50% of human infections, and comprises the most virulent species[3]. In addition, FSSC are usually resistant to azoles *in vitro* and exhibit higher minimum inhibitory concentration for amphotericin B than other *Fusarium* species[32, 33].

The sequencing of the *TEF1α* locus classified the non-FSSC isolated from air into four complexes: FCSC, FFSC, FIESC and FOSC. The FOSC members are of special interest, because they are known as the second most common species in human fusariosis, representing approximately 20% of the cases. Surprisingly, in our study, only one isolate was recovered from the air, and no FOSC was identified from clinical samples. *F*. *oxysporum* isolates have been recovered from several hospital environmental sources, including water[34], shower, sink and bathroom swab[23], but not in the air, suggesting that maybe FOSC is more likely to be recovered from different environmental sources than air.

We used a MLST scheme based on partial sequencing of *TEF1α*, rDNA, *RPB1* and *RPB2* genes [20, 21, 23, 24, 26] for determining the clonality of *Fusarium* isolated from air and blood. New primers sequences were designed to cover regions and gaps of the *RPB2* gene that were not properly sequenced by the primers previously reported. Previous studies reported the molecular relationship between clinical and environmental isolates from hospital water system, hospital surfaces and plumbing drains of external buildings[23, 30, 34, 35]. Revising the recent literature, there are not robust data implicating the air or other environment as potential sources of fusariosis, in hospital settings. Carlesse et al. [36] reported an outbreak of catheter-related fungemia caused by *F*. *oxysporum* but no environmental source was identified. Litvinov et al. [37] described an outbreak of invasive fusariosis in a children´s cancer hospital. They recovered *Fusarium* from the environment (water, air, swabs) of patients’ rooms, but no molecular correlation was done in this study. In the study performed by Edel-Hermann et al. [38] the authors described the clonal linage of *F*. *oxysporum* isolated from the tap water of different French hospitals, but no correlation was established with clinical samples. Short et al. [35] described the occurrence of a clonal distribution of the same sequencing typing of *Fusarium* spp. clinical isolates and in the plumbing drains of hospitals and other facilities.

To our knowledge, our study is the first one describing the identification of two clonal STs: *Fusarium petroliferum* and *Fusarium napiforme* isolated in the air and in blood of patients with fusariosis hospitalized in our hospital. In our study, we found ST1 as the most frequent ST belonging to FSSC (*F*. *petroliphilum*), and ST1 was identified in five of the clinical isolates and in seven isolates from air, indicating a possible environmental source of fusariosis. In addition, one *F*. *napiforme* was isolated from air sampling and the molecular typing showed the same ST for this environmental isolate and for one *F*. *napiforme* recovered from blood (STn1). *F*. *napiforme* was recovered from the patient´s blood culture and from the air of the same room in which the patient was hospitalized, in the hematology unit in 2013[17]. *F*. *napiforme* is a less frequent pathogen and very few cases were previously reported causing keratomycosis[39], hypersensitivity pneumonitis[40], and systemic infection in immunocompromised patients[17, 41].

The molecular typing revealed that *F*. *petroliphilum* and *F*. *napiforme* recovered in the air samplings were related to the ones that caused systemic diseases. In conclusion, systemic fusariosis may occur from the encounter of *Fusarium* spp. present in environmental sources, such as in the air of our hospital, and the susceptible patients hospitalized in BMT and hematology units. This study indicates that there is a need for effective surveillance of hospital environment quality control.

## Supporting information

S1 Table Description of clinical and environmental *Fusarium* spp. isolates according to species, source of isolation and GenBank accession numbers. (DOCX)

S2 Table Climatic conditions and seasons for each air sampling performed for *Fusarium* spp. isolation. (DOCX)

S1 Fig Schematic representation of primers used for DNA amplification in MLST analysis. The long bars represents *TEF1α* (A), rDNA (B), *RPB1* (C) and *RPB2* (D) genes, and the black boxes and numbers above represents the amino acids motifs. The positions of the primers and base pairs (bp) amplified are represented below each gene (right arrow: forward primer; left arrow: reverse primer).
(DOCX)

S2 Fig Phylogenetic tree of *Fusarium fujikuroi* and *Fusarium oxysporum* species complexes. It was generated by maximum likelihood (ML) from 77 –*TEF1α* sequences, 578 characters, percentages of 1,000 bootstrap-replications of MEGA6-maximum likelihood (ML). The tree was rooted with the *F*. *oxysporum* CBS 463.61.
(DOCX)

S3 Fig Phylogenetic tree of *Fusarium solani* species complex. It was generated by maximum likelihood (ML) from 37 –*EF1α* sequences, 570 characters, percentages of 1,000 bootstrap-replications of MEGA6-maximum likelihood (ML). The tree was rooted with *Fusarium staphyleae* NRRL 22316.
(DOCX)

S4 Fig Phylogenetic tree of *Fusarium incarnatum-equiseti* and *Fusarium chlamydoporum* species complexes. It was generated by maximum likelihood (ML) from 43 –*TEF1α* sequences, 556 characters, percentages of 1,000 bootstrap-replications of MEGA6-maximum likelihood (ML). The tree was rooted with *Fusarium sporotrichioides* NRRL 52934. Abbreviations–FIESC: *F*. *incarnatum-equiseti* species complex. FCSC: *F*. *chlamydosporum* species complex.
(DOCX)
