# Supplementary material for: Airborne transmission of invasive fusariosis in patients with hematologic malignancies
Source: PLoS One. 2018 Apr 26;13(4):e0196426. doi: 10.1371/journal.pone.0196426 (PMC5919535; doi:10.1371/journal.pone.0196426)
Supplement: S1 Table — (DOCX) [file pone.0196426.s001.docx]

**S1 Table. Description of clinical and environmental *Fusarium* spp. isolates according to species, source of isolation and GenBank accession numbers.**

| **Species** | **Isolate** | **Source** | **Ward** | **Room** | **Date of isolation** | **GenBank acession number** | | | |
| --- | --- | --- | --- | --- | --- | --- | --- | --- | --- |
|  |  |  |  |  |  | ***TEF1α*** | ***RPB1*** | ***RPB2*** | **Ribosomal DNA** |
| **FFSC** |  |  |  |  |  |  |  |  |  |
| *Fusarium verticillioides* | F19 | Air | BMT | 450 | 12/05/2012 | KU974184 | - | - | - |
| (n=21) | F20 | Air | BMT | 454 | 12/05/2012 | KU974185 | - | - | - |
|  | F21 | Air | BMT | 454 | 12/05/2012 | KU974186 | - | - | - |
|  | F23 | Air | BMT | 458 | 12/05/2012 | KU974188 | - | - | - |
|  | F24 | Air | Hematology | 538 | 12/05/2012 | KU974189 | - | - | - |
|  | F26 | Air | Hematology | 538 | 12/05/2012 | KU974191 | - | - | - |
|  | F28 | Air | Hematology | 538 | 12/05/2012 | KU974193 | - | - | - |
|  | F30 | Air | Hematology | 540 | 12/05/2012 | KU974195 | - | - | - |
|  | F36 | Air | Hematology | 542 | 12/05/2012 | KU974201 | - | - | - |
|  | F37 | Air | Hematology | 542 | 12/05/2012 | KU974202 | - | - | - |
|  | F38-1 | Air | Hematology | 542 | 12/05/2012 | KU974203 | - | - | - |
|  | F39-1 | Air | Hematology | 542 | 12/05/2012 | KU974205 | - | - | - |
|  | F40 | Air | Hematology | 542 | 12/05/2012 | KU974207 | - | - | - |
|  | F42 | Air | Hematology | 542 | 12/05/2012 | KU974209 | - | - | - |
|  | F46 | Air | Hematology | 544 | 12/05/2012 | KU974213 | - | - | - |
|  | F47 | Air | Hematology | 546 | 12/05/2012 | KU974214 | - | - | - |
|  | F48 | Air | Hematology | 546 | 12/05/2012 | KU974215 | - | - | - |
|  | F87 | Air | Hematology | 538 | 03/21/2013 | KU974252 | - | - | - |
|  | F92 | Air | Hematology | 538 | 03/21/2013 | KU974257 | - | - | - |
|  | F95 | Air | Hematology | 542 | 03/21/2013 | KU974260 | - | - | - |
|  | F96 | Air | Hematology | 542 | 03/21/2013 | KU974261 | - | - | - |
| *Fusarium* sp. | F25 | Air | Hematology | 538 | 12/05/2012 | KU974190 | - | - | - |
| (n=17) | F33 | Air | Hematology | 540 | 12/05/2012 | KU974198 | - | - | - |
|  | F65 | Air | Hematology | 540 | 10/10/2012 | KU974231 | - | - | - |
|  | F77 | Air | BMT | 452 | 03/21/2013 | KU974242 | - | - | - |
|  | F80 | Air | BMT | 456 | 03/21/2013 | KU974245 | - | - | - |
|  | F83-1 | Air | BMT | 458 | 03/21/2013 | KU974248 | - | - | - |
|  | F86 | Air | BMT | 460 | 03/21/2013 | KU974251 | - | - | - |
|  | F88 | Air | Hematology | 538 | 03/21/2013 | KU974253 | - | - | - |
|  | F89 | Air | Hematology | 538 | 03/21/2013 | KU974254 | - | - | - |
|  | F97 | Air | Hematology | 542 | 03/21/2013 | KU974262 | - | - | - |
|  | F98 | Air | Hematology | 542 | 03/21/2013 | KU974263 | - | - | - |
|  | F99 | Air | Hematology | 542 | 03/21/2013 | KU974264 | - | - | - |
|  | F100 | Air | Hematology | 542 | 03/21/2013 | KU974164 | - | - | - |
|  | F104 | Air | Hematology | 544 | 03/21/2013 | KU974167 | - | - | - |
|  | F110 | Air | Hematology | 546 | 03/21/2013 | KU974173 | - | - | - |
|  | F114 | Air | Hematology | 548 | 03/21/2013 | KU974177 | - | - | - |
|  | F116 | Air | Hematology | 548 | 03/21/2013 | KU974179 | - | - | - |
| *Fusarium proliferatum* | F13 | Air | Hematology | 548 | 05/03/2012 | KU974180 | - | - | - |
| (n=12) | F15 | Air | Hematology | 546 | 05/03/2012 | KU974182 | - | - | - |
|  | F27 | Air | Hematology | 538 | 12/05/2012 | KU974192 | - | - | - |
|  | F49 | Air | Hematology | 546 | 12/05/2012 | KU974216 | - | - | - |
|  | F81 | Air | BMT | 456 | 03/21/2013 | KU974246 | - | - | - |
|  | F83-2 | Air | BMT | 458 | 03/21/2013 | KU974249 | - | - | - |
|  | F101 | Air | Hematology | 542 | 03/21/2013 | KU974165 | - | - | - |
|  | F105 | Air | Hematology | 544 | 03/21/2013 | KU974168 | - | - | - |
|  | F106 | Air | Hematology | 544 | 03/21/2013 | KU974169 | - | - | - |
|  | F107 | Air | Hematology | 544 | 03/21/2013 | KU974170 | - | - | - |
|  | F108 | Air | Hematology | 544 | 03/21/2013 | KU974171 | - | - | - |
|  | F113 | Air | Hematology | 546 | 03/21/2013 | KU974176 | - | - | - |
| *Fusarium napiforme* | F111 | Air | Hematology | 546 | 03/21/2013 | KU974174 | KU974338 | KU974364 | KU974310 |
| (n=4) | 2008 | **Blood** | Hematology | - | 11/27/2013 | KU974282 | KU974337 | KU974363 | KU974309 |
|  | 2009 | **Blood** | Hematology | - | 11/27/2013 | KU974281 | KU974336 | KU974365 | KU974308 |
|  | 2010 | **Blood** | Hematology | - | 11/27/2013 | KU974280 | KU974335 | KU974366 | KU974307 |
| *Fusarium fujikuroi* | F58 | Air | Hematology | 538 | 10/10/2012 | KU974225 | - | - | - |
| (n=3) | F64 | Air | Hematology | 540 | 10/10/2012 | KU974230 | - | - | - |
|  | F102 | Air | Hematology | 542 | 03/21/2013 | KU974166 | - | - | - |
| *Fusarium pseudocircinatum* | F14 | Air | Hematology | 542 | 05/03/2012 | KU974181 | - | - | - |
| (n=1) |  |  |  |  |  |  |  |  |  |
| *Fusarium subglutinans* | F31 | Air | Hematology | 540 | 12/05/2012 | KU974196 | - | - | - |
| (n=1) |  |  |  |  |  |  |  |  |  |
|  |  |  |  |  |  |  |  |  |  |
| **FSSC** |  |  |  |  |  |  |  |  |  |
| *Fusarium petroliphilum* | F16 | Air | Hematology | 538 | 05/03/2012 | KU974161 | KU974311 | KU974362 | KU974283 |
| (n=19) | F17-1 | Air | BMT | 452 | 03/29/2012 | KU974162 | KU974321 | KU974348 | KU974293 |
|  | F17-2 | Air | BMT | 452 | 03/29/2012 | KU974163 | KU974320 | KU974347 | KU974292 |
|  | F50 | Air | BMT | 450 | 10/10/2012 | KU974217 | KU974319 | KU974346 | KU974291 |
|  | F51 | Air | BMT | 450 | 10/10/2012 | KU974218 | KU974318 | KU974345 | KU974290 |
|  | F52 | Air | BMT | 450 | 10/10/2012 | KU974219 | KU974317 | KU974344 | KU974289 |
|  | F53 | Air | BMT | 452 | 10/10/2012 | KU974220 | KU974327 | KU974340 | KU974299 |
|  | F54 | Air | BMT | 452 | 10/10/2012 | KU974221 | KU974315 | KU974343 | KU974288 |
|  | F55 | Air | BMT | 452 | 10/10/2012 | KU974222 | KU974316 | KU974342 | KU974287 |
|  | F72 | Air | BMT | 452 | 10/10/2012 | KU974238 | KU974314 | KU974341 | KU974286 |
|  | 917 | **Blood** | Hematology | - | 11/09/2007 | KU974273 | KU974326 | KU974350 | KU974298 |
|  | 952 | **Blood** | Hematology | - | 11/28/2007 | KU974267 | KU974312 | KU974349 | KU974284 |
|  | 1196 | **Blood** | BMT | - | 03/29/2008 | KU974268 | KU974313 | KU974354 | KU974285 |
|  | 1549 | **Blood** | Hematology | - | 12/09/2010 | KU974269 | KU974322 | KU974353 | KU974294 |
|  | 1601 | **Blood** | Hematology | - | 12/11/2010 | KU974265 | - | - | - |
|  | 1603 | **Blood** | BMT | - | 03/02/2010 | KU974272 | KU974325 | KU974339 | KU974297 |
|  | 1631 | **Blood** | BMT | - | 07/28/2011 | KU974266 | - | - | - |
|  | 1750 | **Blood** | Hematology | - | 06/03/2011 | KU974271 | KU974324 | KU974352 | KU974296 |
|  | 2020 | **Blood** | Hematology | - | 06/26/2013 | KU974270 | KU974323 | KU974351 | KU974295 |
| *Fusarium keratoplaticum* | 916 | **Blood** | Hematology | - | 10/21/2007 | KU974279 | KU974334 | KU974361 | KU974306 |
| (n=5) | 1103 | **Blood** | Hematology | - | 07/20/2007 | KU974277 | KU974332 | KU974359 | KU974304 |
|  | 1202 | **Blood** | Hematology | - | 03/27/2008 | KU974278 | KU974333 | KU974360 | KU974305 |
|  | 1207 | **Blood** | Hematology | - | 07/23/2008 | KU974275 | KU974329 | KU974357 | KU974301 |
|  | 1372 | **Blood** | BMT | - | 11/16/2008 | KU974276 | KU974331 | KU974358 | KU974303 |
| *Fusarium haematococcum* | F90 | Air | Hematology | 538 | 03/21/2013 | KU974255 | - | - | - |
| (n=2) | F91 | Air | Hematology | 538 | 03/21/2013 | KU974256 | KU974330 | KU974356 | KU974302 |
| *Fusarium* sp. | F112 | Air | Hematology | 546 | 03/21/2013 | KU974175 | - | - | - |
| (n=2) | 1554 | **Blood** | Hematology | - | 01/19/2010 | KU974274 | KU974328 | KU974355 | KU974300 |
|  |  |  |  |  |  |  |  |  |  |
| **FIESC** |  |  |  |  |  |  |  |  |  |
| *Fusariumincarnatum* | F18 | Air | BMT | 452 | 03/29/2012 | KU974183 | - | - | - |
| (n=23) | F22 | Air | BMT | 458 | 12/05/2012 | KU974187 | - | - | - |
|  | F29 | Air | Hematology | 540 | 12/05/2012 | KU974194 | - | - | - |
|  | F32 | Air | Hematology | 540 | 12/05/2012 | KU974197 | - | - | - |
|  | F35 | Air | Hematology | 542 | 12/05/2012 | KU974200 | - | - | - |
|  | F38-2 | Air | Hematology | 542 | 12/05/2012 | KU974204 | - | - | - |
|  | F39-2 | Air | Hematology | 542 | 12/05/2012 | KU974206 | - | - | - |
|  | F41 | Air | Hematology | 544 | 12/05/2012 | KU974208 | - | - | - |
|  | F43 | Air | Hematology | 542 | 12/05/2012 | KU974210 | - | - | - |
|  | F45 | Air | Hematology | 544 | 12/05/2012 | KU974212 | - | - | - |
|  | F56 | Air | Hematology | 538 | 10/10/2012 | KU974223 | - | - | - |
|  | F57 | Air | Hematology | 538 | 10/10/2012 | KU974224 | - | - | - |
|  | F62 | Air | Hematology | 540 | 10/10/2012 | KU974228 | - | - | - |
|  | F63 | Air | Hematology | 540 | 10/10/2012 | KU974229 | - | - | - |
|  | F66 | Air | Hematology | 544 | 10/10/2012 | KU974232 | - | - | - |
|  | F67 | Air | Hematology | 545 | 10/10/2012 | KU974233 | - | - | - |
|  | F69 | Air | Hematology | 548 | 10/10/2012 | KU974235 | - | - | - |
|  | F71 | Air | Hematology | 548 | 10/10/2012 | KU974237 | - | - | - |
|  | F78 | Air | BMT | 456 | 03/21/2013 | KU974243 | - | - | - |
|  | F93 | Air | Hematology | 538 | 03/21/2013 | KU974258 | - | - | - |
|  | F94 | Air | Hematology | 542 | 03/21/2013 | KU974259 | - | - | - |
|  | F109 | Air | Hematology | 546 | 03/21/2013 | KU974172 | - | - | - |
|  | F115 | Air | Hematology | 548 | 03/21/2013 | KU974178 | - | - | - |
| *Fusariumequiseti* | F59 | Air | Hematology | 538 | 10/10/2012 | KU974226 | - | - | - |
| (n=1) |  |  |  |  |  |  |  |  |  |
|  |  |  |  |  |  |  |  |  |  |
| **FCSC** |  |  |  |  |  |  |  |  |  |
| *Fusarium chlamydosporum* | F34 | Air | Hematology | 542 | 12/05/2012 | KU974199 | - | - | - |
| (n=10) | F44 | Air | Hematology | 544 | 12/05/2012 | KU974211 | - | - | - |
|  | F61 | Air | Hematology | 540 | 10/10/2012 | KU974227 | - | - | - |
|  | F68 | Air | Hematology | 546 | 10/10/2012 | KU974234 | - | - | - |
|  | F73 | Air | BMT | 450 | 03/21/2013 | KU974239 | - | - | - |
|  | F74 | Air | BMT | 452 | 03/21/2013 | KU974240 | - | - | - |
|  | F76 | Air | BMT | 452 | 03/21/2013 | KU974241 | - | - | - |
|  | F79 | Air | BMT | 456 | 03/21/2013 | KU974244 | - | - | - |
|  | F82 | Air | BMT | 456 | 03/21/2013 | KU974247 | - | - | - |
|  | F85 | Air | BMT | 458 | 03/21/2013 | KU974250 | - | - | - |
|  |  |  |  |  |  |  |  |  |  |
| **FOSC** |  |  |  |  |  |  |  |  |  |
| *Fusarium oxysporum* | F70 | Air | Hematology | 548 | 10/10/2012 | KU974236 | - | - | - |
| (n=1) |  |  |  |  |  |  |  |  |  |

FCSC: *F. chlamydosporum* species complex*;* FFSC: *F. fujikuroi* species complex*;* FIESC: *F. incarnatum-equiseti* species complex*;* FOSC: *F. oxysporum* species complex; FSSC: *F. solani* species complex*;* BMT: bone marrow transplantation ward.
