# Supplementary material for: Airborne transmission of invasive fusariosis in patients with hematologic malignancies
Source: PLoS One. 2018 Apr 26;13(4):e0196426. doi: 10.1371/journal.pone.0196426 (PMC5919535; doi:10.1371/journal.pone.0196426)
Supplement: S2 Table — (DOCX) [file pone.0196426.s002.docx]

**S2 Table. Climatic conditions and seasons for each air sampling performed for *Fusarium* spp. isolation.**

| **Sampling date** | **Season** | **Maximun temperature (ºC)** | **Maximun humidity (%)** | **Minimun humidity (%)** | ***Fusarium* spp. isolated (n)** |
| --- | --- | --- | --- | --- | --- |
| 03/05/2012 | Summer | 32.4 | 66 | 34 | 4 |
| 03/23/2012 | Autumn | 35.3 | 68 | 76 | 0 |
| 03/29/2012 | Autumn | 24.5 | 72 | 47 | 3 |
| 04/18/2012 | Autumn | 30.4 | 58 | 48 | 0 |
| 05/12/2012 | Autumn | 30.0 | 76 | 49 | 33 |
| 10/10/2012 | Spring | 33.5 | 41 | 30 | 23 |
| 01/03/2013 | Summer | 28.5 | 74 | 69 | 0 |
| 03/15/2013 | Summer | 31.2 | 77 | 61 | 0 |
| 03/21/2013 | Autumn | 24.7 | 98 | 77 | 45 |
