# Supplementary material for: Airborne transmission of invasive fusariosis in patients with hematologic malignancies
Source: PLoS One. 2018 Apr 26;13(4):e0196426. doi: 10.1371/journal.pone.0196426 (PMC5919535; doi:10.1371/journal.pone.0196426)
Supplement: S1 Fig — The long bars represents TEF1α (A), rDNA (B), RPB1 (C) and RPB2 (D) genes, and the black boxes and numbers above represents the amino acids motifs. The positions of the primers and base pairs (bp) amplified are represented below each gene (right arrow: forward primer; left arrow: reverse primer). (DOCX) [file pone.0196426.s003.docx]

**
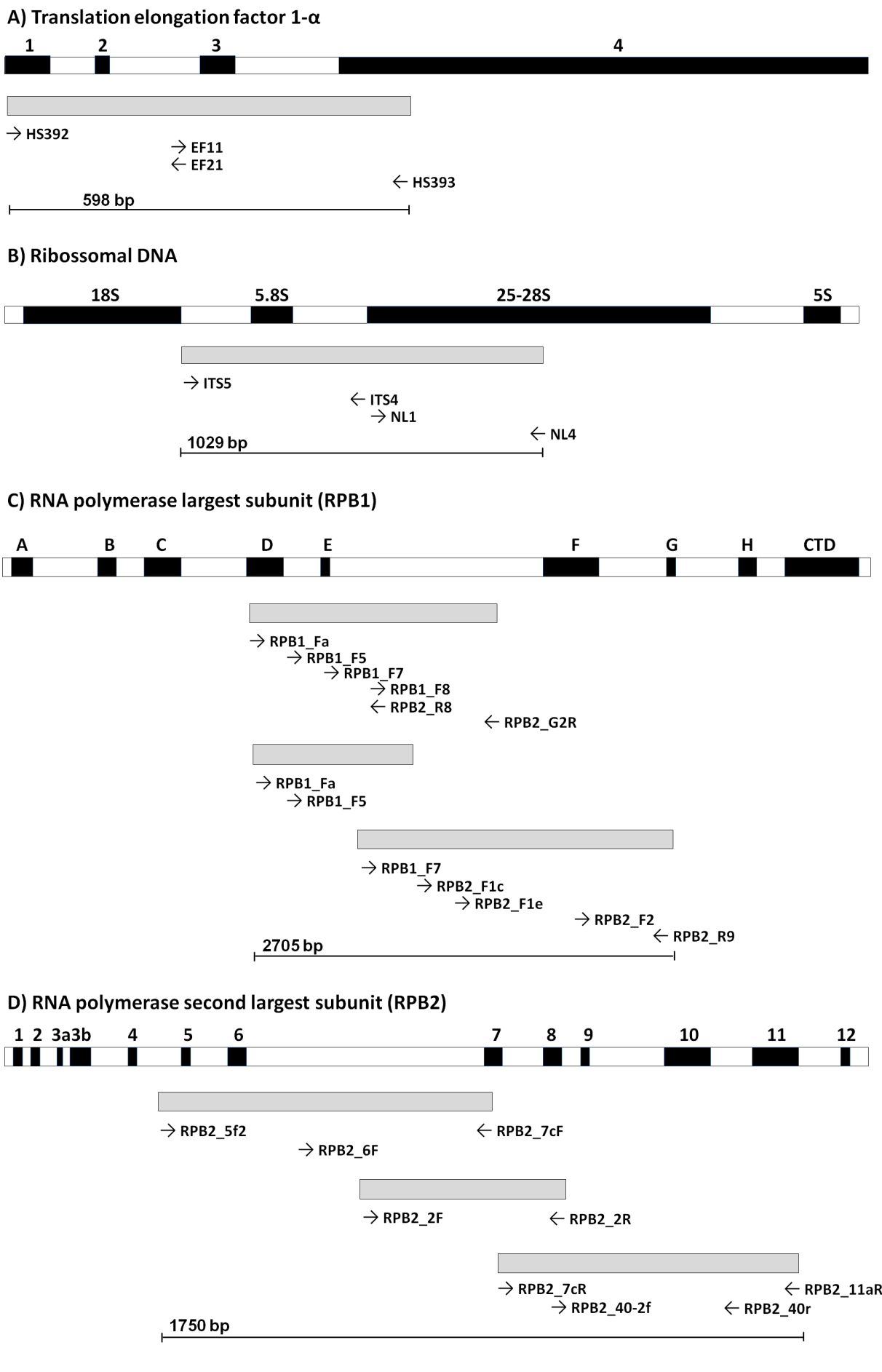
**

**S1 Fig.** **Schematic representation of primers used for DNA amplification in MLST analysis.** The long bars represents *TEF1α* (A), rDNA (B), *RPB1* (C) and *RPB2* (D) genes, and the black boxes and numbers above represents the amino acids motifs. The positions of the primers and base pairs (bp) amplified are represented below each gene (right arrow: forward primer; left arrow: reverse primer)
