# Supplementary material for: Airborne transmission of invasive fusariosis in patients with hematologic malignancies
Source: PLoS One. 2018 Apr 26;13(4):e0196426. doi: 10.1371/journal.pone.0196426 (PMC5919535; doi:10.1371/journal.pone.0196426)
Supplement: S2 Fig — It was generated by maximum likelihood (ML) from 77 –TEF1α sequences, 578 characters, percentages of 1,000 bootstrap-replications of MEGA6-maximum likelihood (ML). The tree was rooted with the F. oxysporum CBS 463.61. (DOCX) [file pone.0196426.s004.docx]

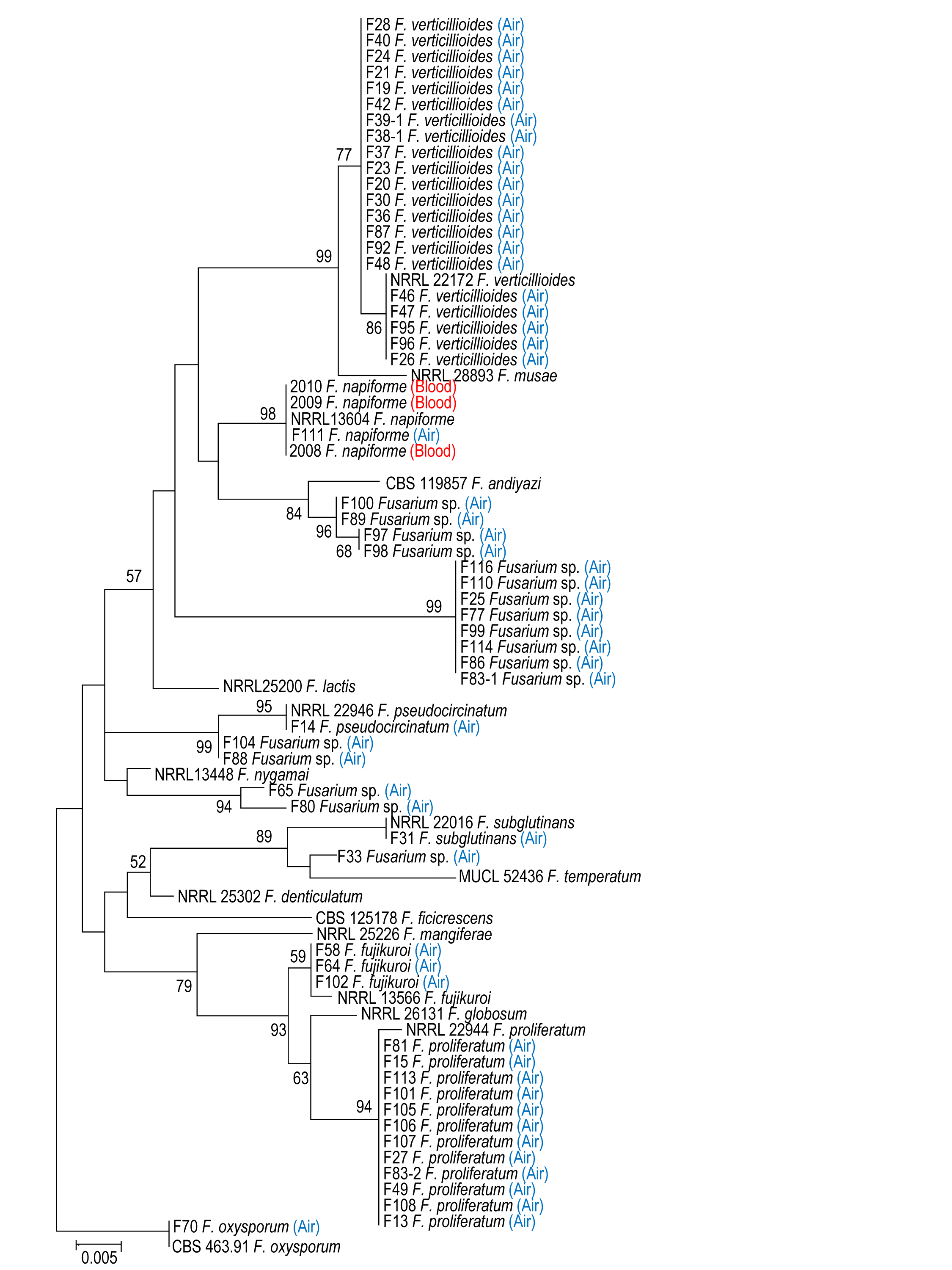


**S2 Fig**. **Phylogenetic tree of *Fusarium fujikuroi* and *Fusarium oxysporum* species complexes.** The tree was generated by maximum likelihood (ML) trees from 77 – *TEF1α* sequences, 578 characters, percentages of 1,000 bootstrap-replications of MEGA6-maximum likelihood (ML). The tree was rooted with the *F. oxysporum* CBS 463.61
