# Supplementary material for: Airborne transmission of invasive fusariosis in patients with hematologic malignancies
Source: PLoS One. 2018 Apr 26;13(4):e0196426. doi: 10.1371/journal.pone.0196426 (PMC5919535; doi:10.1371/journal.pone.0196426)
Supplement: S3 Fig — It was generated by maximum likelihood (ML) from 37 –EF1α sequences, 570 characters, percentages of 1,000 bootstrap-replications of MEGA6-maximum likelihood (ML). The tree was rooted with Fusarium staphyleae NRRL 22316. (DOCX) [file pone.0196426.s005.docx]

**
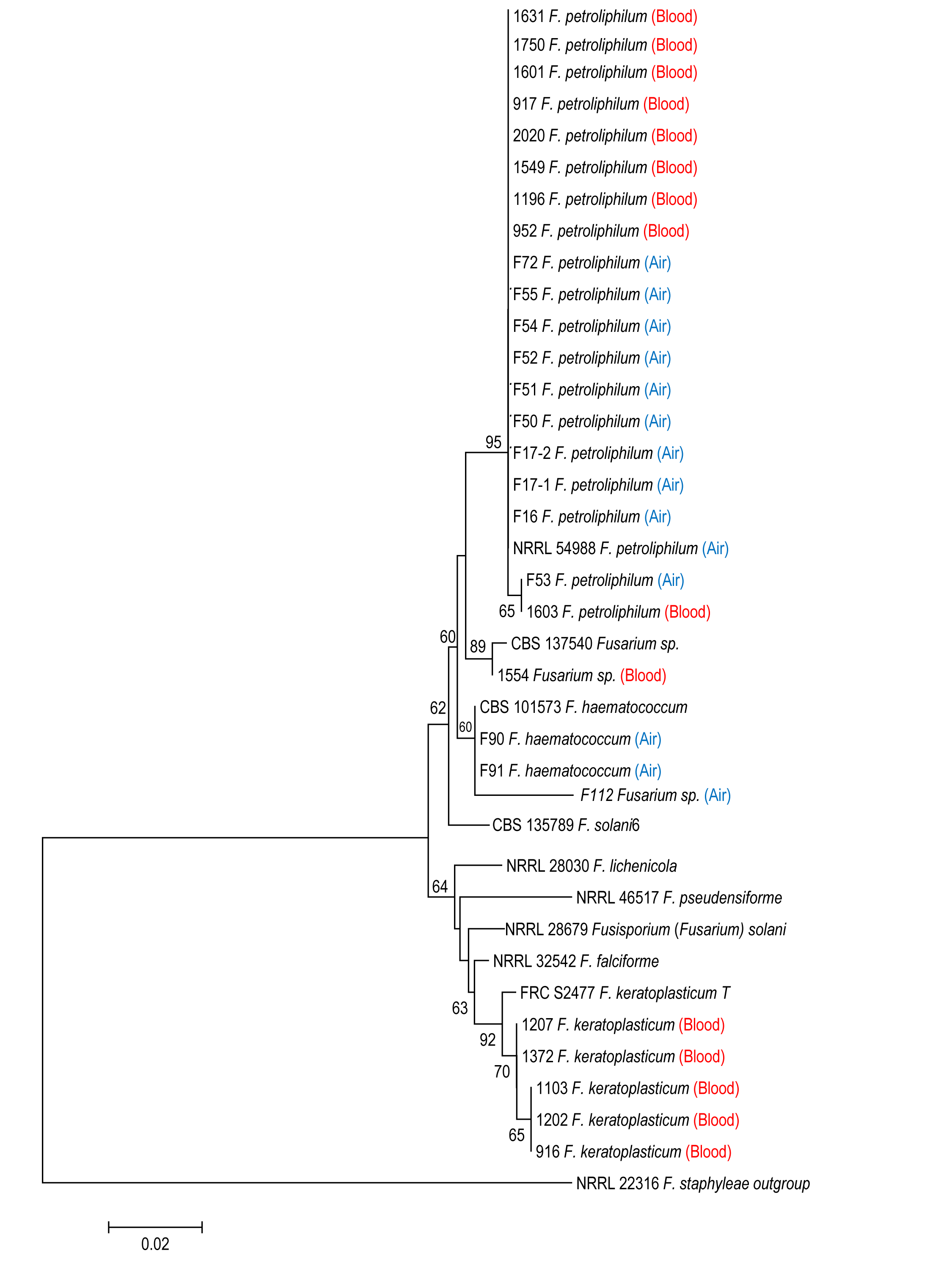
**

**S3 Fig. Phylogenetic tree of *Fusarium solani* species complex.** The tree was generated by maximum likelihood (ML) trees from 37 – *EF1α* sequences, 570 characters, percentages of 1,000 bootstrap-replications of MEGA6-maximum likelihood (ML). The tree was rooted with *Fusarium staphyleae* NRRL 22316.
