# Supplementary material for: Airborne transmission of invasive fusariosis in patients with hematologic malignancies
Source: PLoS One. 2018 Apr 26;13(4):e0196426. doi: 10.1371/journal.pone.0196426 (PMC5919535; doi:10.1371/journal.pone.0196426)
Supplement: S4 Fig — It was generated by maximum likelihood (ML) from 43 –TEF1α sequences, 556 characters, percentages of 1,000 bootstrap-replications of MEGA6-maximum likelihood (ML). The tree was rooted with Fusarium sporotrichioides NRRL 52934. Abbreviations–FIESC: F. incarnatum-equiseti species complex. FCSC: F. chlamydosporum species complex. (DOCX) [file pone.0196426.s006.docx]

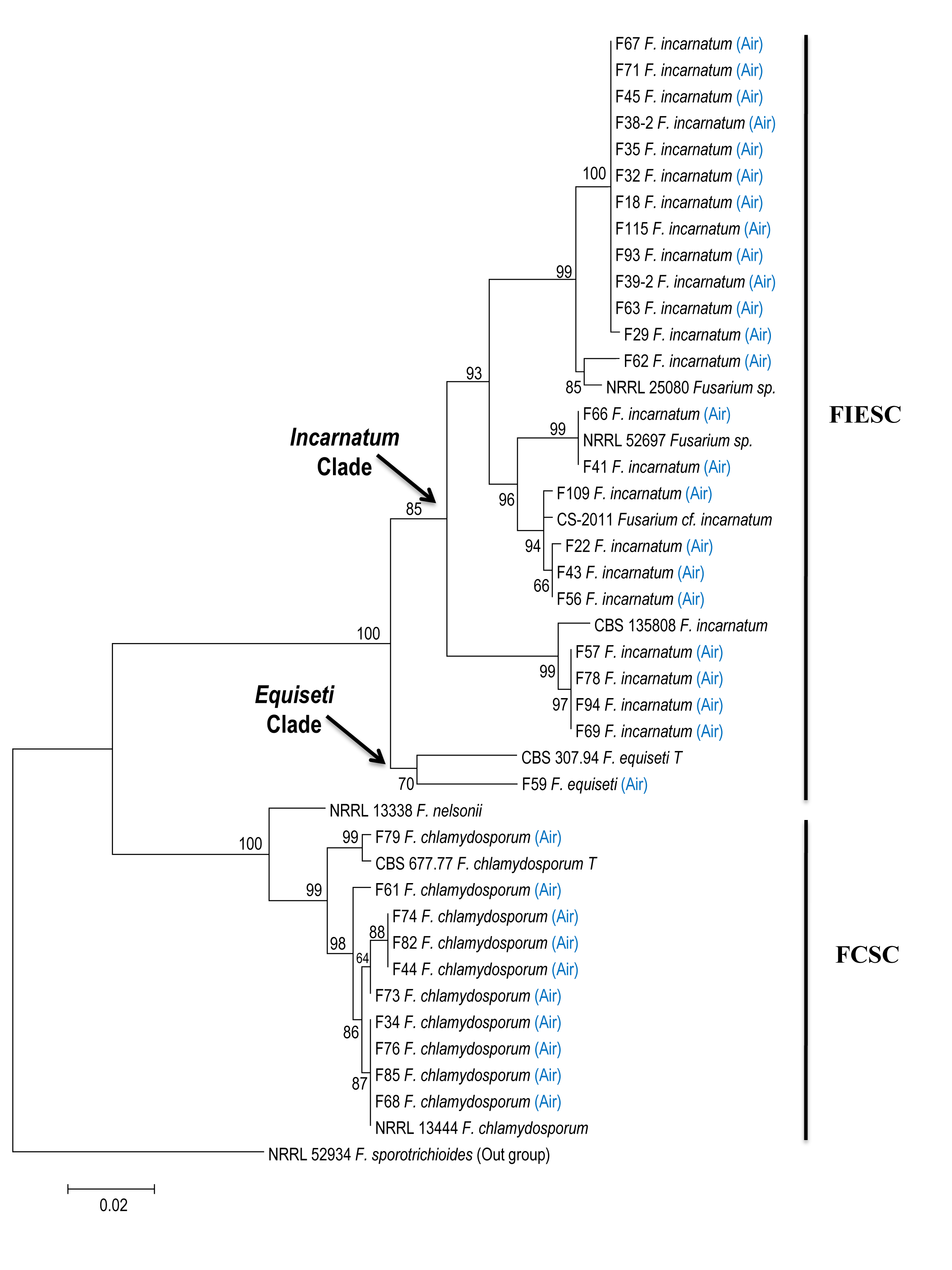


**S4 Fig. Phylogenetic tree of *Fusarium incarnatum-equiseti* and *Fusarium chlamydoporum* species complexes.** The tree was generated by maximum likelihood (ML) trees from 43 – *TEF1α* sequences, 556 characters, percentages of 1,000 bootstrap-replications of MEGA6-maximum likelihood (ML). The tree was rooted with *Fusarium sporotrichioides* NRRL 52934. Abbreviations – FIESC: *F. incarnatum-equiseti* species complex*.* FCSC: *F. chlamydosporum* species complex*.*
